# Comparative Analysis of Infusions and Ethanolic Extracts of *Annona muricata* Leaves from Colima, Mexico: Phytochemical Profile and Antioxidant Activity

**DOI:** 10.3390/life14121702

**Published:** 2024-12-23

**Authors:** Gustavo A. Hernandez-Fuentes, Osiris G. Delgado-Enciso, Edgar G. Larios-Cedeño, Juan M. Sánchez-Galindo, Silvia G. Ceballos-Magaña, Kayim Pineda-Urbina, Mario A. Alcalá-Pérez, Nancy E. Magaña-Vergara, Josuel Delgado-Enciso, Uriel Díaz-Llerenas, Janet Diaz-Martinez, Idalia Garza-Veloz, Margarita L. Martinez-Fierro, Iram P. Rodriguez-Sanchez, Ivan Delgado-Enciso

**Affiliations:** 1 Department of Molecular Medicine, School of Medicine, University of Colima, Colima 28040, Mexico; 1933osiris@gmail.com; 2 State Cancerology Institute of Colima, Health Services of the Mexican Social Security Institute for Welfare (IMSS-BIENESTAR), Colima 28085, Mexico; ehanslarios@gmail.com (E.G.L.-C.); juanmanuelsg02@gmail.com (J.M.S.-G.); 3 Faculty of Chemical Sciences, University of Colima, Coquimatlan 28400, Mexico; kpineda@ucol.mx (K.P.-U.); nancymv@ucol.mx (N.E.M.-V.); 4 Faculty of Sciences, University of Colima, Colima 28045, Mexico; silvia_ceballos@ucol.mx; 5 Molecular Medicine Laboratory, Academic Unit of Human Medicine and Health Sciences, Autonomous University of Zacatecas, Zacatecas 98160, Mexico; marioalcalaperez@uaz.edu.mx (M.A.A.-P.); urieldiazllerenas@gmail.com (U.D.-L.); idaliagv@uaz.edu.mx (I.G.-V.); margaritamf@uaz.edu.mx (M.L.M.-F.); 6 Foundation for Ethics, Education, and Cancer Research of the State Cancer Institute of Colima AC, Colima 28085, Mexico; josuel7@hotmail.com; 7 Research Center in Minority Institutions, Robert Stempel College of Public Health, Florida International University (FIU-RCMI), Miami, FL 33199, USA; jdimarti@fiu.edu; 8 Molecular and Structural Physiology Laboratory, School of Biological Sciences, Autonomous University of Nuevo Leon, San Nicolas de los Garza 66455, Mexico; iramrodriguez@gmail.com; 9 Robert Stempel College of Public Health and Social Work, Florida International University, Miami, FL 33199, USA

**Keywords:** *Annona muricata*, traditional medicine, phytochemistry, antioxidant activity, flavonoids, polyphenols

## Abstract

Background: *Annona muricata* L. (guanabana) leaves are rich in bioactive compounds with potential antioxidant properties. In the state of Colima, both ethanolic extracts and infusions are traditionally used in folk medicine to address various ailments. This study aimed to evaluate and compare the phytochemical composition and antioxidant activities of ethanolic extracts and infusions of *A. muricata* leaves from three geographic regions in Colima, Mexico, with a focus on how geographic origin affects their bioactive properties. Methods: Ethanolic extracts and infusions were prepared from *A. muricata* leaves and analyzed using phytochemical screening; DPPH, total antioxidant capacity (TAC), and total phenolic content (TPC) measurements; and HPLC. TLC was also conducted to examine the presence of specific compounds, such as flavonoids and phenols. Results: Both the ethanolic extracts and infusions contained significant levels of alkaloids, flavonoids, tannins, and phenolic compounds. The infusions demonstrated superior antioxidant capacity, with DPPH inhibition values of 72.5%, 68.3%, and 65.1% in the northern, central, and southern regions, respectively, compared to the ethanolic extracts’ values of 50.3%, 48.9%, and 45.0%. HPLC identified quercetin as a major compound across all samples. Geographically, the northern region exhibited higher concentrations of bioactive compounds, particularly total flavonoid content (TFC) and iron-reducing power (FRPA). Conclusions: Both the ethanolic extracts and infusions of *A. muricata* leaves exhibited significant antioxidant properties, with the infusions showing superior performance. The results suggest that *A. muricata* infusions may have potential applications in managing oxidative stress-related diseases such as cancer and diabetes. Exploring their use in traditional medicine and employing this type of approach can help discern the metabolite profile responsible for these bioactivities. Geographic factors influence the bioactive profile of the plant, and further research is needed to isolate specific bioactive compounds and elucidate their therapeutic mechanisms.

## 1. Introduction

*Annona muricata* L., commonly known as guanabana, belongs to the Annonaceae family, which is part of the order Magnoliales, subclass Magnoliidae, and class Magnoliopsida. This family comprises approximately 2300 species and 130 genera, likely originating from the Americas [1,2]. Members of this family are notable for producing aromatic leaves, fleshy fruits, and commercial-grade wood [2,3]. The leaves of *A. muricata* are simple, smooth-edged, and alternately arranged along the stem.

Guanabana thrives in tropical and subtropical climates, adapting well to diverse soil types. Its natural distribution spans southern Mexico, Central and South America, and the Caribbean islands. In Mexico, over 16,000 tons of guanabana are produced annually, with Nayarit, Colima, and Michoacán being the main producing states [4].

The leaves of *A. muricata* have gained prominence for their medicinal applications, particularly in traditional medicine [5,6]. In Colima, Mexico, they are commonly used to prepare infusions that serve as natural relaxants or remedies for ailments like insomnia, colds, and anxiety [7,8]. Moreover, guanabana leaves are employed in managing chronic conditions such as diabetes and cancer. Despite their widespread use, there is limited scientific information about the bioactive compounds responsible for their reported effects, as well as their safety and appropriate use [9].

From a phytochemical perspective, *A. muricata* leaves are a rich source of bioactive metabolites, including alkaloids, acetogenins, and terpenes [1,2]. Alkaloids such as anonaine, glaucine, and reticuline have demonstrated antimicrobial and anticancer properties [1,10,11]. Acetogenins like annonacin, bullatacin, and muricatacin exhibit potent cytotoxic, antitumor, and antiviral activities. Additionally, terpenes such as β-sitosterol, lupeol, and α-amyrin contribute anti-inflammatory, antioxidant, and antidiabetic effects [6]. However, the concentration of these metabolites can vary significantly due to environmental factors such as climate, soil type, and stressors specific to the region, underscoring the need for localized phytochemical studies [12].

This study aimed to establish a comprehensive phytochemical profile and assess the antioxidant activity of *A. muricata* leaf infusions and ethanol extracts collected from three distinct regions of Colima, Mexico. Using phytochemical screening, spectroscopic methods (UV, IR, and NMR spectroscopy), and chromatographic techniques (TLC and HPLC), we evaluated traditional preparations to identify and confirm the presence of key bioactive compounds. This approach seeks to scientifically validate the traditional uses of *A. muricata*, ensuring its safe and informed application in medicine while addressing the variability in its chemical composition across different regions.

## 2. Materials and Methods

### 2.1. Collection of the A. muricata Leaves

The leaves of *A. muricata* were collected from three representative areas in the state of Colima, Mexico. The first area, known as the North Zone, is located in the municipality of Comala Center (coordinates 19.333692141426315, −103.76188765172623). The second area, known as the Central Zone, is located in the community of “Los Ortices”, municipality of Colima (coordinates 19.122669, −103.734909). The third area, identified as the South Zone, is located in the municipality of Tecomán, Colima, specifically in the “Primaveras community” (coordinates 18°53′55.8″ N 103°52′35.9″ W). The sampling areas were selected based on the diverse biomes and climatic conditions of the state of Colima, Mexico, as described in official reports from the National Institute of Statistics, Geography, and Informatics (INEGI) [13]. The North Zone is characterized by a cooler climate, with warm subhumid conditions and soils predominantly formed from volcanic breccia, which are rich in organic matter. The Central Zone features a temperate to warm climate, with intermediate humidity and soils of volcanic origin that support diverse vegetation. In contrast, the South Zone experiences a hot tropical climate with higher humidity and sandy soils, typical of coastal regions. To ensure the leaves were representative of their respective environments, *A. muricata* specimens were selected from wild populations, avoiding areas with potential contamination such as agricultural fields or plastic waste. Approximately 5 kg of leaves were collected from each zone. The three leaf batches were dried separately by zone in a drying oven with air flow at 30 °C for 72 h. To ensure the correct identification of the species, the collected specimens were compared with an herbarium specimen previously registered under the number MEXU:981827 at the Herbarium of the Museo de Historia Natural of the UNAM (MEXU), which confirmed *A. muricata* as the correct species.

### 2.2. Preparation of Ethanol Extracts

A total of 250 g of dried leaves from each of the three zones, previously crushed in an industrial blender, were weighed. The leaves were placed in separate laboratory glass jars, to which 500 mL of ethanol (Merck, Darmstadt, Germany) was added. The jars were left to rest for 24 h. After the 24 h period, the mixture was filtered using an open-pore filter paper. The filtrates were then concentrated under reduced pressure using a rotary evaporator (IKA RV10, Staufen, Germany), resulting in the respective ethanol extracts. The extract residues obtained were collected and quantified, and the yield was determined (%w/w). The extract was stored at −10 °C until use in an inert atmosphere and according to the protocol of Hernández-Fuentes [14].

### 2.3. Preparation of Infusion of A. muricata Leaves

Following the herbalist’s reference, the infusion was prepared by placing four grams of crushed guanabana leaves (1 leaf = 1 g) leaves in 1 L of water (following the ethnomedical preparation report). The water was brought to a boil, and once it reached 98 °C, the leaves were added. The mixture was allowed to steep in the hot water at 98 °C for 15 min, after which it was filtered. These conditions were reproduced in the laboratory, where the infusion was then subjected to a freezing process followed by lyophilization to obtain a concentrated infusion. The resulting concentrate was stored in amber glass jars under an inert atmosphere in a freezer at −20 °C to reduce degradation until subsequent experiments [7,8].

### 2.4. Qualitative Phytochemical Analysis of A. muricata Leaf Extracts and Infusions

The qualitative phytochemical analysis of both the ethanol and aqueous extracts of *A. muricata* leaves from the three regions was performed following the methods described by Oloya et al. (2021) [15,16]. Tannins were detected using a saturated ferric chloride (FeCl_3_) solution combined with gelatin hydrolysis. Flavonoids were identified through the Shinoda, magnesium, and Salkowski tests. Alkaloids were screened using the Dragendorff, Mayer, and Wager methods. The presence of saponins was determined by performing the hemolysis test with 7% blood agar and observing foam formation in water. Steroids were identified through a reaction with sulfuric acid (H_2_SO_4_) in chloroform, while coumarins were detected by reacting with sodium hydroxide (NaOH). All tests were conducted using a 5 mg/mL concentration of each extract.

### 2.5. Total Flavonoid Content (TFC) in A. muricata Leaf Extract and Infusions

The procedure outlined by Chang et al. in 2020 [17], with modifications from Wakeel et al. in 2019 [18], was adapted for this study. Dilutions of the ethanol extract of *A. muricata* (5 mg/mL in methanol) were prepared, resulting in a final volume of 40 μL. To this, 20 μL of 10% AlCl_3_, 20 μL of 1 M CH_3_CO_2_K, and 380 μL of H_2_O_2_ were added. The reaction mixture was incubated for 30 min, after which the absorbance was measured at 405 nm using a spectrophotometer (BioMate, Thermo Fisher Scientific, Waltham, MA, USA). A standard curve was constructed using quercetin at concentrations of 50, 25, 12.5, and 6.25 μg/mL. The total flavonoid content was expressed as the quercetin equivalent in μg/mg of extract.

### 2.6. Total Antioxidant Capacity (TAC) and Ferric-Reducing Antioxidant Power (FRAP) of A. muricata Leaf Extract and Infusions

The phosphomolybdenum assay [19,20] was used to assess the total antioxidant capacity. In brief, 20 μL of the ethanol extract of *A. muricata* (5 mg/mL in methanol) was mixed with 180 μL of phosphomolybdenum reagent (0.6 M sulfuric acid, NaH_2_PO_4_ 28 nM, 4 mM ammonium molybdate) and incubated at 95 °C for 90 min in a water bath. After incubation, the absorbance was measured at 630 nm using a spectrophotometer (BioMate, Thermo Fisher Scientific, Waltham, MA, USA). Ascorbic acid was used as a positive control. The antioxidant capacity was calculated using the following formula: % antioxidant capacity = [1 − (OD of the sample/OD of the control)] × 100.

The ferric-reducing antioxidant power (FRAP) was determined using the potassium ferrocyanide–ferric chloride method [21], with slight modifications [22,23,24]. Briefly, 40 μL of the ethanol extract of *A. muricata* (5 mg/mL in methanol) was mixed with 50 μL of phosphate buffer (0.2 M, pH 6.6) and 50 μL of 1% potassium ferrocyanide, and incubated at 50 °C for 20 min. Afterward, 50 μL of 10% trichloroacetic acid was added, and the mixture was centrifuged at 3000 rpm for 10 min. The supernatant was collected, and 33.3 μL of 0.1% FeCl3 was added. The absorbance was then measured at 630 nm using a spectrophotometer (BioMate, Thermo Fisher Scientific, Waltham, MA, USA). The reducing power was calculated using the following formula: % reducing power = [1 − (OD of the sample/OD of the control)] × 100.

### 2.7. Determination of Total Phenolic Content (TPC)

The total phenolic content was evaluated using the Folin–Ciocalteu (FC) reagent method, as described by Jafri et al. (2017) [25], with modifications for microplate analysis based on the approach by Wakeel et al. (2019) [18]. A stock solution of the FC reagent was prepared by diluting the 2N reagent to 1N using deionized distilled water. In the assay, 20 μL of the sample extract was added to each well of a 96-well plate, followed by the addition of 90 μL of the FC reagent. After a 5 min incubation, 90 μL of a 20% sodium bicarbonate solution was introduced to each well. The plate was then incubated at room temperature for 90 min. Gallic acid was used to create a standard calibration curve with final concentrations of 1000, 500, 200, 100, 40, 20, 10, and 5 μg/mL (y = 0.4925x − 0.4479, r^2^).

### 2.8. Spectroscopic Analysis of A. muricata Leaf Extracts and Infusions

UV, FTIR, and NMR spectroscopic analyses were carried out on both the ethanol and infusion extracts of *A. muricata* leaves from the three different regions. UV spectra were recorded with an Evolution 300 spectrophotometer (Thermo Fisher Scientific, Waltham, MA, USA) in a methanol (MeOH) solution. FTIR measurements were taken using a Varian 660-IR spectrophotometer. ^1^H NMR spectra were collected on a Bruker NMR spectrometer (Bruker, Leipzig, Germany) with frequencies of 400 MHz. All extracts were dissolved in DMSO-d6 (Sigma Aldrich, St. Louis, MO, USA) as the solvent. The chemical shifts were reported in δ (ppm), and the coupling constants (J) were presented in Hz. These chemical shifts were compared with those of previously isolated compounds from *A. muricata* leaves (see Supplementary Table S1).

### 2.9. Chromatography Analysis by TLC and HPLC

TLC analysis was carried out using the methodology described by Gwatidzo et al. [24] with slight modifications. Five 5 × 5 cm TLC plates (Silica gel 60 F254, Supelco, Bellefonte, PA, USA) were prepared with pencil lines drawn 0.5 cm from one edge. Extract samples, prepared at a concentration of 5.0 mg/mL, were applied (2 μL) onto the pencil lines using micropipettes. For comparison, three standards were used: quercetin (Essential Nutrition, Monterrey, Mexico), 4-methylumbelliferone, and anthrone (Sigma Aldrich, St. Louis, MO, USA), each at a concentration of 1 mg/mL. The plates were placed in a development chamber and exposed to a trial solvent, either chloroform (CHCl_3_) or a mixture of CHCl_3_ and methanol (MeOH). After development, the plates were removed, and the solvent front was marked with a soft pencil. The plates were examined under UV light at 254 and 365 nm for visualization. To reveal the compounds, the plates were sprayed with ceric sulfate and 1% ferric chloride (for phenol detection). The chromatograms were then photographed, and the retention factor (Rf) was calculated using the following formula: Rf = distance traveled by the spot/distance traveled by the solvent front. All TLC analyses were performed in triplicate for consistency.

To establish the chromatographic profile of the *A. muricata* extracts and infusions from three distinct regions, a polyphenol-based analytical approach was utilized, adapting the methodologies described by Sakakibara (2003) [26], V. Cijo George (2015) [27] and Hernandez-Fuentes (2020) [28]. High-performance liquid chromatography (HPLC) was performed using a Waters e2695-Alliance system equipped with a photodiode array detector (DAD, model 2998, Waters Corporation, Milford, MA, USA). The separation of compounds was carried out with an XBRIDGE C18 column (150 mm × 4.6 mm, 3.5 µm particle size, Waters Corporation, Milford, MA, USA). The mobile phase consisted of acetonitrile and water acidified with 0.05% formic acid, and a gradient program was designed to optimize the separation of the target compounds. The gradient was set as follows: from 0 to 10 min, 20% acetonitrile and 80% acidified water; from 10 to 40 min, a gradual increase in acetonitrile content to 80%, which was held constant until 50 min; and from 50 to 60 min, a return to the initial ratio of 20% acetonitrile and 80% acidified water. The flow rate was maintained at 0.8 mL/min, with an injection volume of 20 µL, and the column temperature was kept at 35 ± 5 °C. UV–VIS spectral scans were recorded within a range of 200–400 nm, and the total run time was 40 min.

The reference standards used for identification and quantification included gallic acid (GA), cinnamic acid (CA), anthrone (ANT), quercetin (Q), and 4-methylumbelliferone (4-MU), all sourced from Sigma-Aldrich (St. Louis, MO, USA). The reagents, including HPLC-grade acetonitrile (Sigma-Aldrich, USA) and Milli-Q water, were prepared for the mobile phase, samples, and standards. Samples were filtered prior to injection using 0.22 µm Spritzen-Syringe filters (Spritzen, Germany), and analytical weights were measured with a high-precision balance (Ohaus Corporation, Parsippany, NJ, USA). The ethanolic extracts and infusions were prepared from *A. muricata* leaves collected in three regions. The ethanolic extracts were tested at concentrations from 500 to 5000 ppm, while infusions were analyzed at concentrations from 500 to 3000 ppm.

### 2.10. Anti-Browning Assay of Freshly Cut Apple Slices

The procedure followed the methodology of Lee et al. (2023) [29], with slight modifications. Sample Preparation: Apples (Malus domestica ‘Starking Delicious’) of uniform ripeness were purchased from a local market in Obregón, Colima, Mexico. The apples were sliced into consistent pieces, with dimensions of 1.5 ± 0.1 cm × 1.5 ± 0.1 cm × 0.3 ± 0.05 cm. Three slices were placed in 5 cm diameter glass dishes. Sample Treatment: Each slice was treated with one of the following: a control solution (ethanol/water), a 0.5 mg/mL vitamin C solution (positive control), or ethanolic extracts or infusions of *A. muricata* at 0.5 mg/mL (ethanol/water). The dishes were incubated at 20 °C. Observations: Browning effects were monitored at 0, 12, 24, 36, and 48 h (the last time point of the experiment). Browning Color Measurement: At five time points (0, 12, 24, 36, and 48 h), photographs of the apple slices were taken using an iPhone 14 Pro. The color changes (a*, b*, and L*) were analyzed using CorelDRAW version 2024 (V. 25.0) software, averaging the measurements from five different areas of each slice. Color was quantified using the CIE color system: a* (redness and greenness), b* (yellowness and blueness), and L* (lightness). The total color difference (∆E) was calculated using the following formula: (∆E)^2^ = (a − a_initial)^2^ + (b − b_initial)^2^ + (L − L_initial)^2^ [29].

## 3. Results

The *A. muricata* study allowed for the exploration of the moisture content and extraction yields from leaves collected across different regions of Colima, Mexico, highlighting slightly regional variations in these parameters. First, the moisture content of the freshly harvested *A. muricata* leaves was determined based on the weight loss after a drying procedure at 30 °C for 72 h. For the North Zone, the initial weight of 5 kg was reduced to 4.63 kg after drying, resulting in a moisture content of 7.4%. For the Central Zone, the initial weight of 5 kg was reduced to 4.61 kg, corresponding to a moisture content of 7.8%. Finally, for the South Zone, the initial weight of 5 kg was reduced to 4.62 kg, indicating a moisture content of 7.6%. These values align with those of previous studies on drying procedures for *A. muricata*.

The ethanolic extracts obtained from the leaves had a semi-solid, green-black appearance. The yield for each zone was as follows: 10.8 g (yield: 4.32%) for the North Zone, 12.5 g (yield: 5.00%) for the South Zone, and 12.16 g (yield: 4.86%) for the Central Zone. As for the infusions, each of the three zones yielded a solid that was brown in color, with the following yields: 100 mg (yield: 0.004%) for the North Zone, 80 mg (yield: 0.0032%) for the South Zone, and 98 mg (yield: 0.00392%) for the Central Zone.

The phytochemical screening of the ethanolic extracts of *A. muricata* leaves (Table 1) revealed the presence of various bioactive compounds in different concentrations. The alkaloid tests, using Mayer’s, Dragendorff’s, and Wagner’s reagents, all indicated a moderate presence of alkaloids, which was confirmed by consistent positive reactions across all three tests. Similarly, the flavonoid content was tested using the Shinoda test, which also demonstrated a moderate concentration, suggesting that flavonoids are a significant component of this extract. Further analysis showed the presence of chalcones and quinones, indicating these compounds’ potential role in the extracts’ bioactivity. However, coumarins were completely absent, as indicated by a negative result in the NaOH test. For tannins, the gelatin hydrolysis test revealed a moderate concentration, implying that tannins are present but not in high quantities. The ferric chloride test for phenols also indicated a moderate amount, adding to the extracts’ profile of phenolic compounds. Conversely, lignans were absent, as shown by a negative reaction in the phenol test.

On the other hand, the *A. muricata* leaf infusions (Table 1) showed the presence and absence of various compounds. The alkaloid tests, which would typically involve Mayer’s, Dragendorff’s, and Wagner’s reagents, do not produce results in this analysis, so it remains unclear whether alkaloids were present in significant quantities. Similarly, the screening for flavonoids, using the Shinoda and Salkowski tests, lacked definitive results, leaving the flavonoid content undetermined in this case.

However, the analysis indicated the presence of chalcones and quinones, although details on their concentration were not provided. The NaOH test for coumarins showed a negative result, confirming that coumarins are absent in the infusion. The tests for tannins (using gelatin hydrolysis), phenols (using ferric chloride), and lignans also lacked definitive results, so their presence or absence remained undetermined.

Comparing the phytochemical screening results of the ethanolic extracts and the infusions of *A. muricata* leaves revealed significant differences in the presence of secondary metabolites. The ethanolic extracts showed a moderate presence of alkaloids, flavonoids, tannins, and phenols, whereas these same groups were not found in the infusion, suggesting that ethanol is more effective at solubilizing these compounds than water. Both extraction methods agree on the absence of coumarins, confirmed by the negative result in the NaOH test [30]. Additionally, both methods detected the presence of chalcones and quinones, though without concentration details for the infusions. It is important to denote that the preliminary phytochemical screening, while useful, could lead to potential misinterpretations if considered alone. To ensure accuracy, these findings should be confirmed with more advanced techniques. However, due to the cost of these methods, preliminary phytochemical screening remains a valuable and practical starting point for identifying potential bioactive compounds [31].

### 3.1. Antioxidant and Phenolic Concentration of Ethanolic Extracts and Infusions of A. muricata Leaves

Table 2 presents a comparative analysis of the bioactive compounds in and antioxidant properties of the ethanolic extracts and infusions from the three different zones (A, B, and C). The key metrics evaluated included total flavonoid content (TFC), iron-reducing power (FRPA), total antioxidant capacity (TAC), total polyphenol content (TPC), and DPPH antioxidant capacity. These measurements provide insight into the variations in phytochemical composition and antioxidant activity between the two extraction methods, highlighting the differences between zones and offering a deeper understanding of the bioactive potential of the samples. The results suggested that the infusions generally exhibited superior antioxidant properties and higher concentrations of key compounds compared to the ethanolic extracts.

For the ethanolic extracts, the total flavonoid content (TFC) was highest in Zone A (80.40 ± 4.68 µg/mg), followed by Zone B (73.74 ± 7.22 µg/mg) and Zone C (63.70 ± 4.23 µg/mg). This shows a gradual decrease in flavonoid content from Zone A to Zone C. The iron-reducing power (FRPA) was also highest in Zone A (41.25 ± 4.40%), followed by Zone B (40.61 ± 7.86%) and Zone C (36.20 ± 4.89%), indicating a slightly lower reduction in iron-reducing capacity in the latter zones. The total antioxidant capacity (TAC) was highest in Zone B (34.45 ± 7.54%), followed by Zone C (30.23 ± 8.21%) and Zone A (24.57 ± 5.12%). The total polyphenol content (TPC), expressed in gallic acid equivalents (GAE), was highest in Zone B (57.23 ± 7.56 µg/mg), followed by Zone A (47.89 ± 2.54 µg/mg) and Zone C (45.11 ± 4.56 µg/mg). The DPPH antioxidant capacity was highest in Zone B (70.31 ± 13.24%), followed by Zone A (50.68 ± 5.85%) and Zone C (48.46 ± 17.76%).

The total flavonoid content (TFC) was highest in the infusions from Zone C (93.70 ± 1.63 µg/mg), followed by the infusions from Zone A (90.40 ± 10.68 µg/mg) and Zone B (83.74 ± 5.23 µg/mg). This indicates a marked increase in flavonoid content compared to the ethanolic extracts. The iron-reducing power (FRPA) in the infusions was significantly higher than in the ethanolic extracts, with Zone B showing the highest reduction (75.62 ± 4.36%), followed by Zone C (70.58 ± 5.30%) and Zone A (69.61 ± 1.23%). The total antioxidant capacity (TAC) was also higher in the infusions, with Zone C showing the highest value (65.23 ± 1.29%), followed by Zone A (64.57 ± 1.27%) and Zone B (54.57 ± 1.28%). The total polyphenol content (TPC) was highest in Zone A (80.08 ± 1.56 µg/mg), followed by Zone B (79.10 ± 2.36 µg/mg) and Zone C (69.56 ± 4.56 µg/mg), which were notably higher than those of the ethanolic extracts. The DPPH antioxidant capacity was also higher in the infusions, with Zone B showing the highest percentage of inhibition (90.13 ± 10.25%), followed by Zone C (78.45 ± 10.64%) and Zone A (70.64 ± 4.23%).

When contrasting the results from the preliminary phytochemical screening with the findings from the antioxidant and bioactive compound assays, several key observations emerged. Interestingly, the Salkowski test for flavonoids showed the presence of chalcones in the ethanolic extracts of the North and South Zones, and quinones in all zones for both extract types. This could help explain the higher flavonoid content observed in these regions, which may be linked to their antioxidant activity, as reflected by the TAC and DPPH results [32,33,34]. The significant presence of flavonoids in the ethanolic extracts, especially from the North and South Zones, aligns with their superior antioxidant properties in the TFC assay [19,35].

In contrast, phenolic compounds, detected by the ferric chloride test, were present in moderate amounts in both the ethanolic extracts and infusions across all zones. This finding corresponds with the TPC results, where a higher polyphenol content was noted in the infusions, suggesting that the infusion method may better extract polyphenols. This supports the higher antioxidant activity observed in the infusions, particularly in the TAC and DPPH assays. Moreover, compounds like coumarins, tannins, and lignans were absent in both the ethanolic extracts and infusions. This suggests that these compounds did not significantly contribute to the observed antioxidant activity in the extracts produced using either extraction method. In summary, while the ethanolic extracts show notable levels of alkaloids and flavonoids, the infusions demonstrated superior overall antioxidant capacity. The higher levels of polyphenols and better DPPH activity in the infusions indicate that this extraction method might enhance the release of certain bioactive compounds, particularly polyphenols and flavonoids, which contribute to stronger antioxidant properties [32].

### 3.2. Spectroscopic Analysis of Ethanolic Extracts and Infusions of A. muricata Leaves

Regarding the UV spectrum, maxima were observed at 210, 236, and 289 nm for all three batches of ethanolic extracts, while for the infusions, similar maxima were observed at 280 and 360 nm. These peaks suggest the presence of aromatic compounds, likely flavonoids and polyphenols, in both the ethanolic extracts and the infusions, with the differences in the exact wavelengths, indicating the influence of the extraction method on the composition of the bioactive compounds.

In the IR spectra, the ethanolic extracts (Figure S1) displayed a fingerprint region with maxima at 1610 and 1735 cm^−1^, which are likely indicative of C=C stretching vibrations of aromatic rings and C=O stretching (carbonyl groups), respectively. These bands suggest the presence of flavonoids or other polyphenolic compounds, which are typical in plant extracts. Additionally, signals at 2857, 2927, and 2972 cm^−1^ correspond to C-H stretching vibrations from methylene (-CH_2_) and methyl (-CH_3_) groups, which are commonly found in the alkyl chains of the phytochemicals present in the extract [36].

Furthermore, a broad band at 3338 cm^−1^, extending from 3042 to 3600 cm^−1^, is indicative of O-H stretching vibrations, commonly associated with hydroxyl groups in phenolic compounds, flavonoids, and other hydroxylated molecules. The broadness of this band suggests strong hydrogen bonding interactions, which are characteristic of phenolic compounds [37,38]. The intensity of the band at 80.0% transmittance suggests a high concentration of these hydroxylated groups in the ethanolic extracts [36]. This confirms the presence of bioactive polyphenols and flavonoids, which contribute to the antioxidant properties observed in the previous assays.

In the infusions, the pattern was very similar; however, the intensity of the O-H stretching band was much more intense, reaching 60% transmittance, which could suggest a higher concentration of hydroxylated compounds, such as polyphenols and flavonoids, compared to the ethanolic extracts. The increased intensity of this band in the infusions may indicate that the infusion method facilitates the extraction of these compounds more efficiently, potentially leading to a greater presence of bioactive hydroxylated groups. This observation further supports the superior antioxidant properties observed in the infusions, as hydroxylated compounds are known for their antioxidant activity (Figure S1).

**Figure 1:**
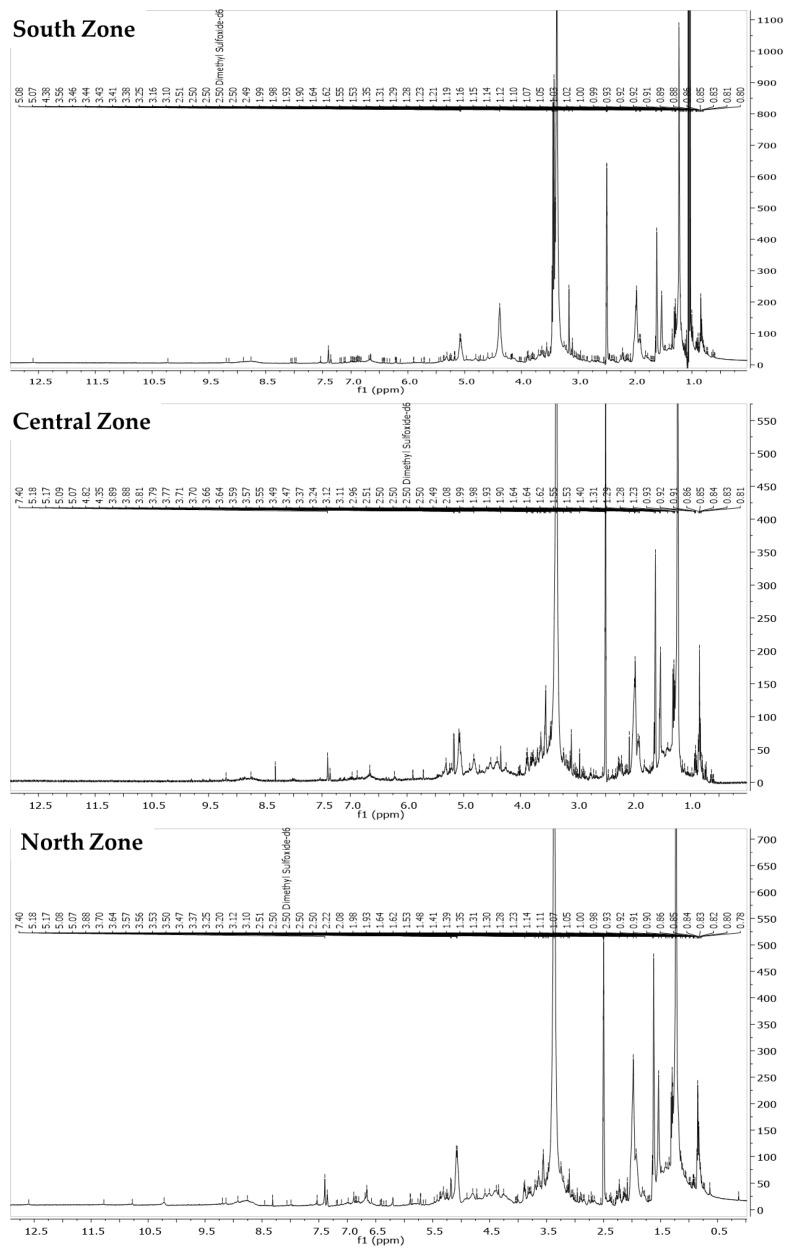
Comparison of ^1^H NMR (400 MHz, DMSO-d6) profiles of ethanolic extracts from the North, Central, and South Zones produced under specific extraction conditions.

Upon analyzing the ^1^H NMR spectra (400 MHz, DMSO) (Figure 1) of the ethanolic extracts from the North, Central, and South Zones, several similarities and key patterns were observed across the samples, suggesting the presence of common bioactive compounds, in line with previous results from the preliminary phytochemical screening as well as the antioxidant capacity, UV, and IR analyses. In the case of the North Zone, the spectrum showed a series of signals between δ 12.60 and δ 0.13 ppm, with significant peaks at δ 9.19, 8.92, 8.45, 7.53, 7.40, 7.35, 6.66, 6.42, 6.36, and 3.47. These signals are characteristic of aromatic protons, which are typically found flavonoids and polyphenolic compounds, such as flavones and chalcones [39,40,41]. The broad shift at δ 12.60 could correspond to protons from hydroxyl or carbonyl groups, which are common in polyphenolic compounds.

The Central Zone spectrum presented signals that overlap with those from the North Zone, such as peaks at δ 9.19, 8.75, 7.40, 7.36, 6.97, 6.88, and 5.71. These signals also indicate aromatic protons from flavonoids and polyphenolic compounds. The presence of the peak at δ 9.19 suggests the possible presence of a hydroxylated flavonoid or chalcone-type compound. Additionally, the signals between δ 5.09 and δ 5.18 ppm likely correspond to methylene or methine protons associated with these bioactive compounds. On the other hand, the South Zone spectrum showed several similarities with that of the other zones, with prominent signals at δ 12.60, 9.20, 8.89, 8.76, 8.06, and 7.53, which also suggest the presence of flavonoids and polyphenolic compounds. The abundance of signals between δ 3.50 and δ 0.60 ppm is characteristic of aliphatic protons, possibly from sugars or other components of the extracts. This suggests that the ethanolic extracts from all three zones contained polyphenolic compounds, particularly flavonoids and chalcones, which are known for their antioxidant properties. The signals in the mentioned regions aligned with the findings from the other chemical and biological tests, indicating that these compounds may be responsible for the bioactive properties observed in the extracts.

**Figure 2:**
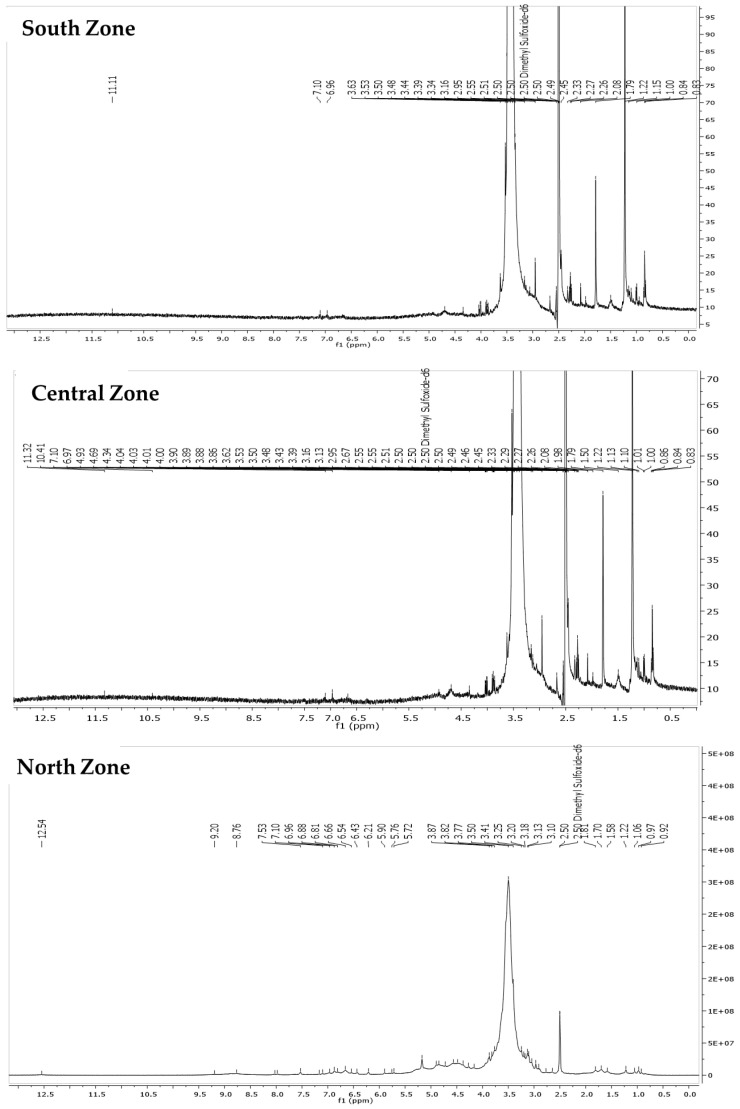
Comparison of ^1^H NMR (400 MHz, DMSO-d6) profiles of infusions from the North, Central, and South Zones produced under specific extraction conditions.

The ^1^H NMR spectra (400 MHz, DMSO) of the infusion extracts from the North, Central, and South Zones exhibited approximately 80–90% similarity (Figure 2), with shared signals in both the aromatic (δ 9.20–7.10 ppm) and aliphatic (δ 3.50–0.60 ppm) regions, indicating the presence of common bioactive polyphenolic compounds across the samples. In the North Zone, key signals appeared at δ 12.54, 9.20, 8.76, 8.03, 7.98, 7.53, 7.17, 7.10, 6.96, 6.88, 6.81, 6.66, 6.54, 6.43, and 6.21 ppm, which are typical of flavonoids and chalcones. The aliphatic region showed additional signals at δ 3.50–0.92 ppm, suggesting the presence of sugars or other components. The Central Zone displayed overlapping signals, including δ 12.62, 9.19, 8.76, 7.98, 7.96, 7.52, 7.10, 6.96, and 6.87 ppm, reinforcing the presence of similar polyphenolic compounds. The aliphatic region also shared signals between δ 3.91 and δ 0.93 ppm, indicating common components with the North Zone [42,43]. The South Zone spectrum was almost identical to that of the North Zone, with nearly identical chemical shifts, confirming the consistency of the polyphenolic compounds in these two zones.

### 3.3. Chromatographic Analysis Using TLC and HPLC

Initially, a general analysis was conducted using thin-layer chromatography (TLC) with phenolic compound standards, including quercetin, 4-methylumbelliferone, cinnamic acid, gallic acid, and anthrone (Figure S2). These standards were selected as comparison patterns due to their structural diversity and their classification as phenolic derivatives, aiming for the potential qualitative identification of similar compounds. The analysis revealed that both the guanabana infusion and the guanabana extracts exhibited similar profiles. However, specific responses to revealing agents, such as ferric chloride (for phenols) and ceric sulfate (a general reagent), highlighted unique characteristics [44,45,46]. Specifically, spots with a retention factor (Rf) of 0.90 were observed, which exhibited absorption at 365 nm in all three batches of the ethanolic extract (with reddish to wine-colored hues). Additionally at this wavelength, traces of a compound with an Rf value similar to that of 4-ML (0.85) were detected in the ethanolic extract batches.

These findings suggested that the extracts and infusions might be rich in phenolic derivatives, including coumarins, flavonoids, and simple phenols. To confirm this hypothesis, high-performance liquid chromatography coupled with a diode-array detector (HPLC-DAD) was employed, with a wavelength scan ranging from 200 to 400 nm. As a first step, a standard chromatogram profile was developed using the standards previously utilized in the TLC analysis. These standards were chosen for their phenolic nature and structural diversity, providing a robust framework for identifying compounds of interest. Quercetin was selected as the representative for flavonoids, as it is commonly found in phenolic-rich plant extracts. Similarly, 4-methylumbelliferone was included as a model compound for coumarins, while cinnamic acid and gallic acid were used to represent hydroxycinnamic and hydroxybenzoic acids, respectively. Anthrone was also incorporated, as it reflects a unique structural class of phenolic derivatives often associated with plant secondary metabolism.

**Figure 3:**
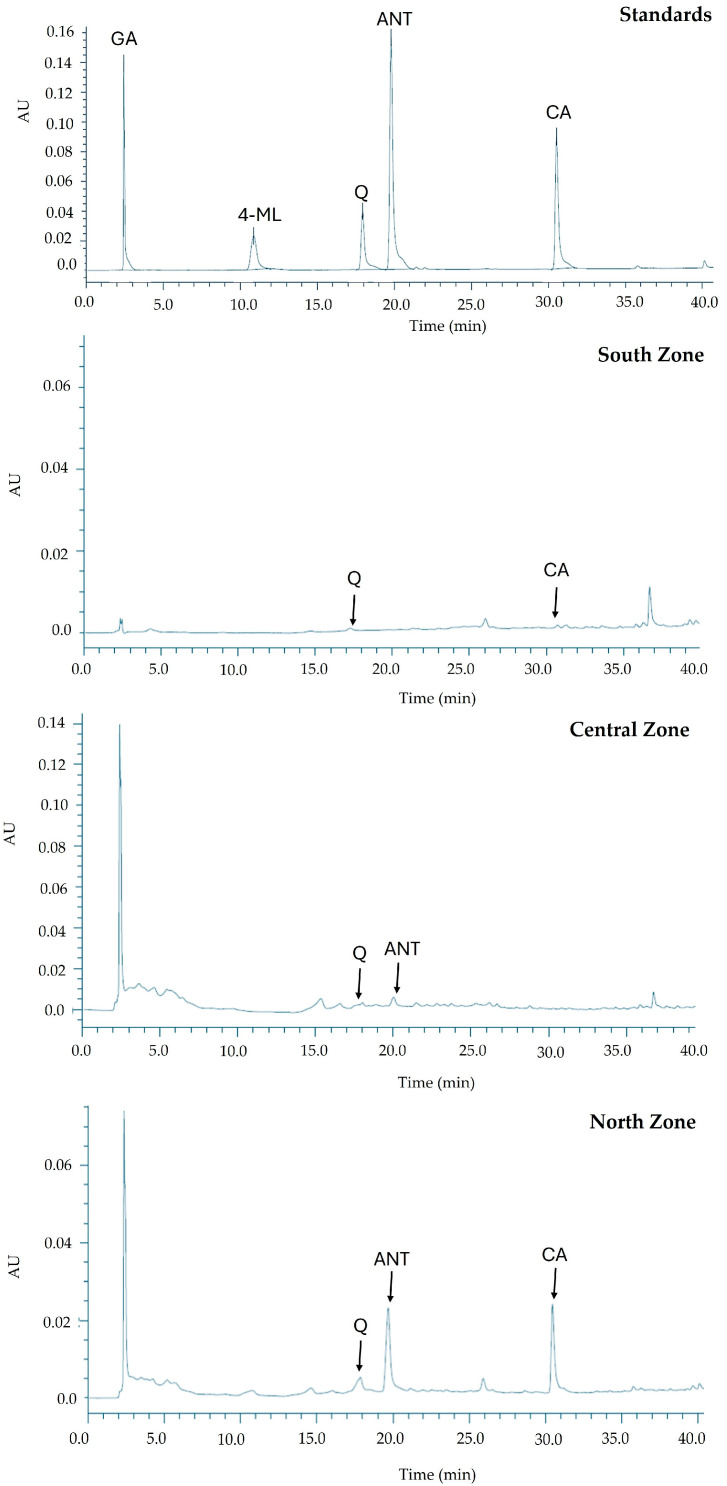
Comparison of HPLC chromatograms (290 nm) for the ethanolic extracts analyzed at 1000 ppm from the three collection zones: North, Central, and South. The chromatogram of the standards includes gallic acid (GA; Rt: 2.385 min), cinnamic acid (CA; Rt: 30.795 min), anthrone (ANT; Rt: 20.000 min), quercetin (Q; Rt: 17.955 min), and 4-methylumbelliferone (4-ML; Rt: 10.908 min). All the standards (quercetin (Q), 4-methylumbelliferone (4-ML), anthrone (ANT), gallic acid (GA), and cinnamic acid (CA)) were injected at a concentration of 100 ppm each.

The chromatograms in Figure 3 provide a comparison of the chemical profiles obtained from the ethanolic extracts of plants collected from the North, Central, and South Zones, analyzed at 1000 ppm, against the chromatogram of known standards. The standards exhibited distinct peaks corresponding to specific compounds: gallic acid (GA) at a retention time (Rt) of 2.385 min, 4-methylumbelliferone (4-ML) at 10.908 min, quercetin (Q) at 17.955 min, anthrone (ANT) at 20.000 min, and cinnamic acid (CA) at 30.795 min. In the South Zone extract, quercetin (Q) and cinnamic acid (CA) were detectable, but their peak intensities were much lower compared to the standards, indicating reduced concentrations of these compounds. The Central Zone extract revealed peaks corresponding to quercetin (Q) and anthrone (ANT), suggesting the presence of these flavonoids in moderate quantities. The North Zone extract displayed peaks for quercetin (Q), anthrone (ANT), and cinnamic acid (CA), with slightly higher intensities than the other zones, particularly for cinnamic acid.

Importantly, quercetin (Q) was consistently detected across all three extracts under the employed extraction conditions, regardless of the collection zone. This consistency suggests that quercetin could be used as a reliable internal marker or control in future studies assessing the chemical profiles of these ethanolic extracts based on the conditions in the areas where they were collected, and considering the specific climatic conditions of each zone. Overall, the chromatograms highlighted the variations in the presence and concentrations of bioactive compounds among the three zones, which could reflect differences in environmental factors, plant metabolism, or extraction efficiency. The North Zone appeared to have the most diverse and prominent chemical profile, as evidenced by the presence of multiple key compounds.

**Figure 4:**
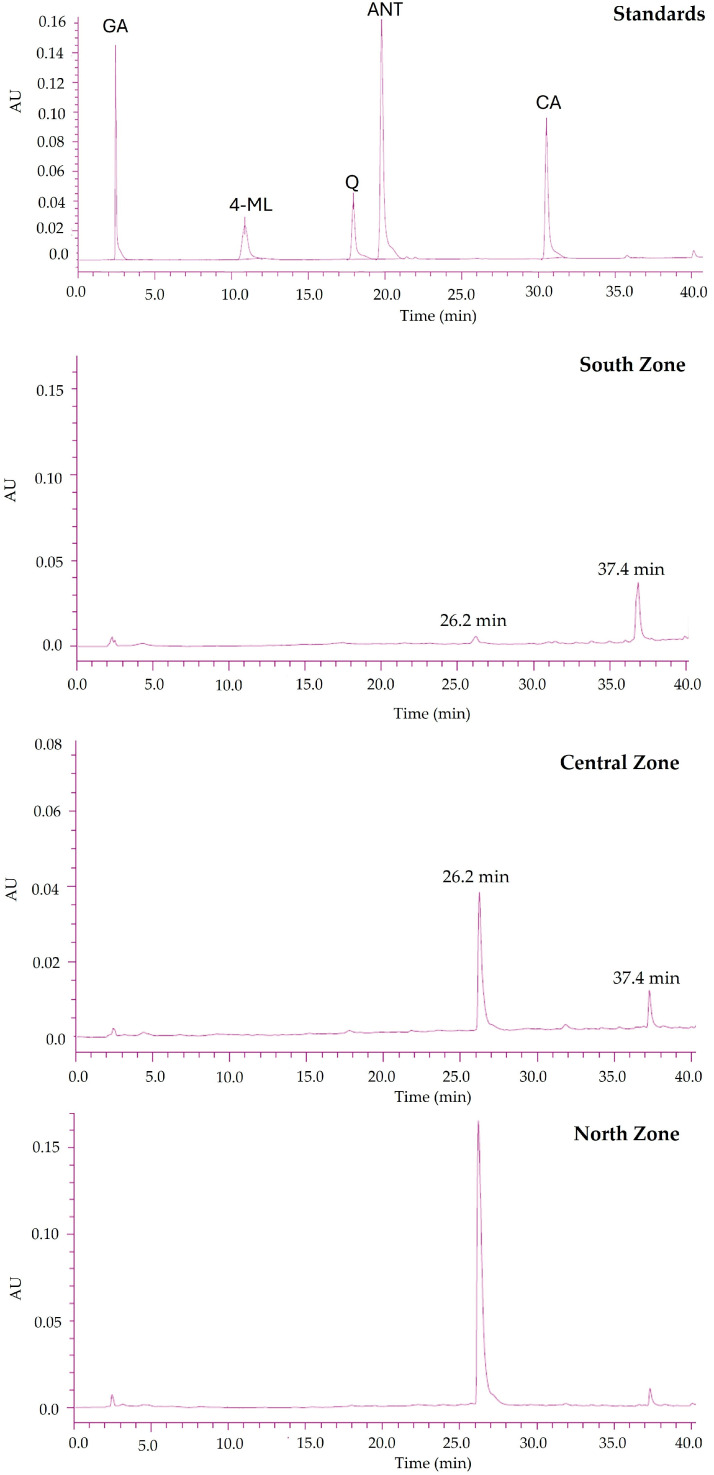
Comparison of HPLC chromatograms (290 nm) for the infusions analyzed at 2000 ppm from the three collection zones: North, Central, and South. The chromatogram of the standards includes gallic acid (GA; Rt: 2.385 min), cinnamic acid (CA; Rt: 30.795 min), anthrone (ANT; Rt: 20.000 min), quercetin (Q; Rt: 17.955 min), and 4-methylumbelliferone (4-ML; Rt: 10.908 min). All the standards (quercetin (Q), 4-methylumbelliferone (4-ML), anthrone (ANT), gallic acid (GA), and cinnamic acid (CA)) were injected at a concentration of 100 ppm each.

In the analyzed HPLC chromatograms of the infusions (Figure 4), a prominent peak with a retention time of 26.2 min was observed, showing an area under the curve (AUC) of 3,564,296 in the extract from the Central Zone. This peak was also detected in the extracts from the North and South zones, albeit with slightly different intensities. It was determined that the AUC of the peak at 26.2 min in the North Zone was approximately 3,800,000, while in the South Zone, it was approximately 3,300,000. This peak exhibited UV absorptions at 236.8 nm and 291.4 nm.

A second significant peak was identified at a retention time of 37.4 min, with UV absorptions proportional to the first peak. Based on these proportions, it was calculated that the AUC of the second peak was 900,000 for the Central Zone, approximately 950,000 for the North Zone, and 850,000 for the South Zone.

Although these peaks did not match any of the employed standards (gallic acid, 4-methylumbelliferone, quercetin, anthrone, and cinnamic acid), their UV absorption characteristics and retention times suggested that they may correspond to derivatives of known metabolites or unique compounds present in the infusions from these geographic zones.

Further studies are required to confirm the identity of these peaks and determine whether they represent previously described metabolites or novel compounds with potential bioactive properties. Advanced analytical techniques, such as mass spectrometry and nuclear magnetic resonance spectroscopy, will be necessary for precise structural identification.

### 3.4. Anti-Browning Assay of the Infusion and Ethanolic Extract of A. muricata

**Figure 5:**
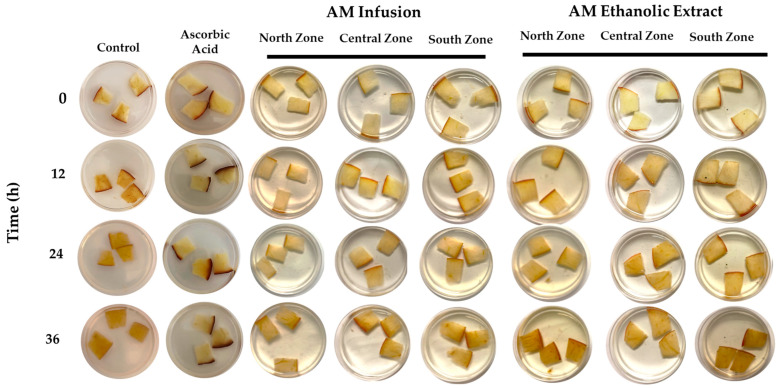
Schematic representation of the ani-browning assay results for the infusions and ethanolic extracts of *A. muricata*.

**Figure 6:**
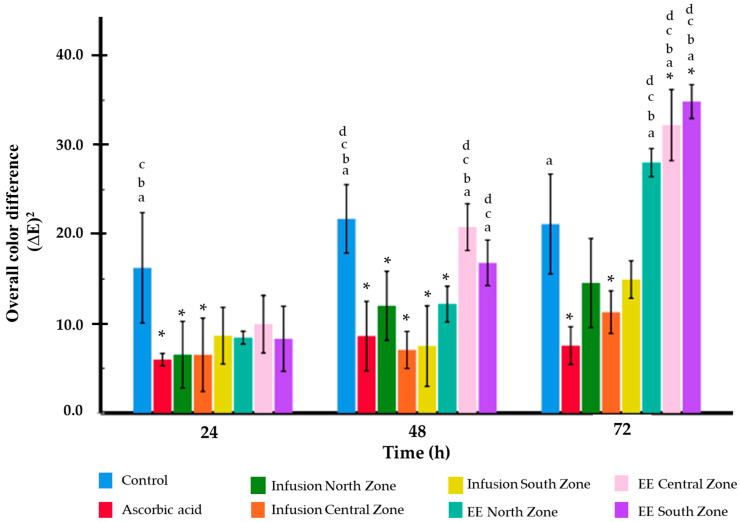
The overall color difference (∆E^2^) in the anti-browning assay of apple slices treated with the *A. muricata* ethanolic extracts (EEs) and infusions was evaluated. All the treatments were tested at a concentration of 0.5 mg/mL, with distilled water as the control and ascorbic acid (0.5 mg/mL) as a reference. The statistical analysis was performed using Tukey’s post hoc test: * statistically significant difference (*p* < 0.05) compared to the control; ^a^ statistically significant difference (*p* < 0.05) compared to ascorbic acid; ^b^ statistically significant difference (*p* < 0.05) compared to the infusion from the North Zone; ^c^ statistically significant difference (*p* < 0.05) compared to the infusion from the Central Zone; and ^d^ statistically significant difference (*p* < 0.05) compared to the infusion from the South Zone.

The analysis of the antioxidant capacity of the treatments, measured through the browning of freshly cut apple slices, revealed significant differences between the evaluated groups (Figure 5). Ascorbic acid, used as the positive control, consistently showed the lowest increase in ∆E, indicating superior antioxidant efficacy across all the evaluated time points. In Figure 6, it is possible to observe the results of the anti-browning assay. At 24 h, the infusions from the North (6.54 ± 1.87) and Central (6.52 ± 2.06) Zones exhibited statistically similar performances to that of ascorbic acid (5.98 ± 0.35), suggesting notable antioxidant capacities. In contrast, the South zone infusion (8.66 ± 1.59) and the ethanolic extracts, particularly those from the Central (9.94 ± 1.61) and South (8.31 ± 1.82) Zones displayed higher ∆E values, reflecting lower antioxidant efficacies. At 48 h, ascorbic acid (8.61 ± 1.94) maintained an excellent antioxidant capacity. The infusions from the Central (7.06 ± 1.03) and South (7.50 ± 2.25) Zones also demonstrated low browning values, remaining statistically similar to that of ascorbic acid (*p* > 0.05).

However, the ethanolic extracts from the North (12.19 ± 1.00) and especially the Central (20.81 ± 1.31) Zones exhibited significantly higher ∆E values, suggesting reduced antioxidant capacities or potential prooxidant effects. At 72 h, the browning was notably higher in the ethanolic extracts, with the Central Zone extract (32.23 ± 1.99) showing the highest ∆E, indicating a pronounced prooxidant trend. Conversely, ascorbic acid (7.55 ± 1.06) and the infusions from the North (14.56 ± 2.49) and South (14.94 ± 1.05) Zones continued to demonstrate moderate antioxidant capacity, maintaining lower browning levels compared to most of the other ethanolic extracts. These findings highlight the variability in antioxidant capacity among the treatments, with ascorbic acid remaining the most effective.

## 4. Discussion

This study highlighted the significant variations in the phytochemical composition and antioxidant properties of ethanolic extracts and infusions of *A. muricata* leaves from three different geographic zones. These findings underscore the importance of considering environmental factors, such as soil composition, climate, and harvesting conditions, which have a substantial impact on the bioactive potential of plant extracts. The results suggest that *A. muricata* can be a valuable source of antioxidants, with both ethanolic extracts and infusions demonstrating potent antioxidant activity, although differences in their bioactive profiles were observed across the geographic regions.

The phytochemical analysis showed that the ethanolic extracts contained notable amounts of alkaloids, flavonoids, tannins, and phenols, while the infusions displayed a superior antioxidant capacity, as indicated by higher DPPH, TAC, and TPC results. These results align with those of previous studies, which demonstrated that water-based extractions typically enhance the release of polyphenols and related compounds, significantly contributing to antioxidant activity [47]. The enhanced DPPH inhibition in the infusions suggests that water, with its higher polarity, facilitates the extraction of hydrophilic antioxidant compounds, in contrast to ethanol, which is more efficient at extracting non-polar compounds [18].

Interestingly, the presence of chalcones and quinones in both the ethanolic extracts and infusions may help explain the high flavonoid content and antioxidant activity observed in this study. These compounds are known for their redox properties, which neutralize free radicals and reduce oxidative stress [48,49,50]. Similarly, the stronger antioxidant capacity of the infusions in the DPPH assay suggests that aqueous extraction is more effective in isolating certain bioactive compounds, which may be responsible for this enhanced activity. The fact that the infusions consistently showed superior antioxidant capacity compared to the ethanolic extracts could be attributed to the ability of water to extract a broader range of hydrophilic antioxidants, particularly polyphenols, which have been linked to strong antioxidant properties [39,51]. Previous research has indicated that the higher polarity of water compared to ethanol allows for more efficient extraction of bioactive compounds, especially those that are more soluble in aqueous environments [52]. Additionally, studies have shown that aqueous alcoholic extraction solvents, such as ethanol–water mixtures, can be highly effective in extracting a range of bioactive compounds, including flavonoids [53,54,55]. This difference in polarity may explain why infusions, which are typically prepared by steeping plant materials in hot water, tend to preserve and concentrate bioactive compounds like polyphenols, flavonoids, and other antioxidants, potentially leading to more potent antioxidant activity [56]. This finding is consistent with those of studies on other plants, where aqueous extracts demonstrated superior antioxidant and anti-inflammatory properties compared to alcoholic extracts [57].

Geographic variability was another notable finding, as the extracts from the North Zone exhibited higher concentrations of bioactive compounds compared to those from the Central and South Zones. This geographic difference is consistent with the findings from similar studies on *A. muricata* and other medicinal plants, where environmental factors were shown to influence the phytochemical profile and bioactive properties of plant extracts [58]. It is important to highlight a significant finding regarding the variations observed in the TAC and TPC values between the ethanolic extracts and infusions of *A. muricata*. These differences may be linked to environmental factors such as soil composition, climate, and geographic characteristics of the regions studied. The state of Colima, Mexico, exhibits notable diversity in these aspects due to its unique geological formation. For instance, the North Zone (Comala, Colima, Mexico) is characterized by higher altitudes (approximately 750 m above sea level), a warm sub-humid climate with summer rains, and volcanic breccia soils, which may influence the synthesis and concentration of specific metabolites. In contrast, the South Zone (Tecoman, Colima, Mexico) features sandy soils and a warmer, more humid climate, which could affect both the production of metabolites and their extraction efficiency in ethanolic and aqueous preparations. These environmental variations likely explain the trends observed, although further studies are needed to isolate and characterize these metabolites to better understand the mechanisms driving their production [59]. Also, the higher total flavonoid content (TFC) and iron-reducing power (FRPA) in the extracts from the North Zone could reflect better-developed protective mechanisms in plants from this region, possibly related to the increased production of secondary metabolites in response to environmental stressors, such as varying temperatures and soil types [58]. Furthermore, it is worth noting that the trees selected for sampling were verified to be over two years old and to possess buttresses, following the guidelines from the consulted manuals [60,61]. This was performed to ensure that their growth was not compromised and to establish optimal management practices for the species. While the consistency of the leaves was standardized by selecting those with a similar structure and without damage, tree age and growth stage may still influence the phytochemical profile and antioxidant activity. Older trees might produce different concentrations of secondary metabolites compared to younger ones, potentially affecting the antioxidant capacity of the extracts and infusions [62,63,64].

The HPLC analysis provided a more detailed chemical profile of the extracts, showing that quercetin was a constant marker in all the collected fractions, suggesting that this flavonoid is a prominent compound in *A. muricata*, regardless of the geographic region. This aligns with the findings of previous studies that identified quercetin as a major bioactive compound in *A. muricata* [6]. The consistent presence of quercetin across all the samples suggests that this compound could be used as an internal marker for future studies on the chemical characterization of *A. muricata* extracts. Moreover, the differences in peak intensity observed across the regions indicate that environmental factors may influence the concentration of this bioactive compound. The spectroscopic analysis further confirmed the presence of flavonoids and phenolic compounds, supporting the antioxidant properties observed in the assays. The UV absorption peaks identified in the HPLC analysis correspond to compounds known for their antioxidant potential, further reinforcing the bioactive capabilities of *A. muricata*. These findings are in agreement with those of studies that have shown that flavonoids and phenolic compounds are major contributors to the antioxidant activities of plant extracts (Rohman et al., 2017 [65]).

Despite these promising results, the study had several limitations. One limitation was the lack of specific quantitative data for certain metabolites, such as alkaloids, in the infusions, which could provide a more comprehensive understanding of the bioactive components. Moreover, the study only focused on three geographic regions, which may not be representative of the entire range of environmental variability in *A. muricata* cultivation areas. Future studies should aim to isolate and characterize the specific bioactive compounds responsible for the observed antioxidant activities and explore their mechanisms of action. Additional bioactivity assays, such as anti-inflammatory or antimicrobial tests, could also complement the antioxidant evaluations to provide a more holistic assessment of the therapeutic potential of *A. muricata*. One last limitation of this study was the lack of standardization in the particle size of the crushed *A. muricata* leaves. While traditional medicinal practices guided the preparation of the plant material, where leaves were triturated manually using a mortar or grinder to produce a coarse, non-uniform powder, the particle size was not explicitly controlled. This variability in particle size may influence the interaction between the plant material and solvents, potentially affecting the extraction efficiency [66]. In future studies, we plan to establish a standardized method for determining particle size to ensure more consistent and optimized extraction conditions.

The findings of this study have significant implications for the future development of *A. muricata* (guanabana) as a natural therapeutic agent. Given the potent antioxidant properties demonstrated by both the ethanolic extracts and infusions, particularly the latter, the results suggest that *A. muricata* infusions could be explored as a complementary treatment for various diseases related to oxidative stress, such as cancer and diabetes. Antioxidants play a crucial role in neutralizing free radicals, which are involved in the development of these conditions [33,57,67,68]. Additionally, the potential anti-inflammatory and anticancer properties of *A. muricata* have been reported in several studies, with compounds like quercetin exhibiting anticancer activity by inducing apoptosis in cancer cells and inhibiting tumor growth. The infusion’s enhanced antioxidant capacity could further contribute to reducing hyperglycemia and improving insulin sensitivity in diabetic patients, as oxidative stress is known to exacerbate insulin resistance. These findings provide a scientific basis for the potential therapeutic use of *A. muricata* infusions in the management of oxidative stress-related diseases, encouraging future research into its clinical applications.

It would be beneficial to incorporate standardized HPTLC methods for the quantification and detection of alkaloids and flavonoids, as this technique offers greater accuracy and reliability. Overcoming challenges related to the availability of alkaloid standards will be essential for advancing this approach. The adoption of HPTLC could serve as a more precise method for quality control in the analysis of plant-based extracts, complementing conventional techniques and providing more robust results in phytochemical studies [56]. Regarding the results for the ethanolic extracts, in which quercetin was proposed as a probable metabolite marker, further studies could focus on confirming its role as a reliable marker across different environmental conditions and plant growth stages. Investigating quercetin’s consistency and variability in various extracts from different seasons or collection zones will provide a better understanding of its stability and reliability as an internal marker in future research. Finally, in this study, we compared the traditional infusion method with ethanolic extracts prepared using conventional techniques. It would be interesting to standardize an extraction method for the components from guanabana leaves in future studies to better evaluate both extraction methods and to establish additional assays such as such as proximate analyses, mineral content determination, toxicity tests, bioactivity screenings, or stability analyses in order to extrapolate the results to other bioassays.

One of the key strengths of this study is the comprehensive approach to assessing the antioxidant properties of *A. muricata* through multiple methods, including DPPH, TAC, and TPC assays, along with HPLC and TLC analyses. The inclusion of infusions as well as ethanolic extracts offers a broader perspective on the potential bioactivity of *A. muricata*, considering that both forms of extraction are commonly used in traditional medicine. Furthermore, the study provides valuable insights into how geographic origin can affect the chemical composition and bioactivity of plant extracts, which has significant implications for the standardization of plant-based therapies.

Another strength of the study is its contribution to the growing body of research on *A. muricata* as a source of natural antioxidants. While several studies have investigated the phytochemical composition and medicinal properties of *A. muricata* (Nayak et al., 2021 [69]), few have focused on the influence of geographic origin on its bioactive properties, making this study an important addition to the literature.

## 5. Conclusions

In conclusion, this study demonstrated that both ethanolic extracts and infusions of *A. muricata* leaves contain significant levels of bioactive compounds with antioxidant properties. The geographic variability observed in the phytochemical composition of the extracts highlights the importance of considering environmental factors when evaluating the therapeutic potential of medicinal plants. The findings suggest that both extraction methods are effective for obtaining antioxidant-rich extracts, with infusions showing superior antioxidant activity. Future research should focus on isolating and characterizing the specific bioactive compounds responsible for these effects and exploring their mechanisms of action to better understand the therapeutic potential of *A. muricata*.

## Figures and Tables

**Table 1:** Phytochemical screening of *A. muricata* leaf extracts and infusions from different zones.

*A. muricata*	Ethanolic Extracts			Infusions		
Test	North Zone	Central Zone	South Zone	North Zone	Central Zone	South Zone
Mayer (Alkaloids)	+++	++	+++	+	+	+
Dragendorff (Alkaloids)	+++	+++	+++	+	+	+
Wagner (Alkaloids)	+++	+++	+++	+	+	+
Shinoda (Flavonoids)	+++	++	+++	+++	++	+++
Salkowski (Flavonoids)	Chalcones	Quinones	Quinones	Chalcones	Quinones	Quinones
NaOH Test (Coumarins)	-	-	-	-	-	-
Gelatin Hydrolysis (Tannins)	-	-	-	-	-	-
Ferric Chloride (Phenols)	++	++	++	++	++	++
Phenol Test (Lignans)	-	-	-	-	-	-

+++ = appreciable amount (positive within 5 min); ++ = moderate amount (positive after 5 min but within 10 min); + = trace amount (positive after 10 min but within 15 min); - = completely absent. Concentration of extract was 5 mg/mL.

**Table 2:** Comparative analysis of bioactive compounds and antioxidant properties in ethanolic extracts and infusions from different zones.

Test	Ethanolic Extract			Infusion		
	North Zone	Central Zone	South Zone	North Zone	Central Zone	South Zone
TFC	80.40 ± 4.68	73.74 ± 7.22	63.70 ± 4.23	90.40 ± 10.68	83.74 ± 5.23	93.70 ± 1.63
FRPA	41.25 ± 4.40	40.61 ± 7.86	36.2.0 ± 4.89	61.89 ± 1.23	62.23 ± 4.36	58.56 ± 5.30
TAC	24.57 ± 5.12	34.45 ± 7.54	30.23 ± 8.21	64.57 ± 1.27	54.57 ± 1.28	65.23 ± 1.29
TPC	47.89 ± 2.54	57.23 ± 7.56	45.11 ± 4.56	80.08 ± 1.56	79.10 ± 2.36	69.56 ± 4.56
DPPH	50.68 ± 5.85%	70.31 ± 13.24%	48.46 ± 17.76%	70.64 ± 4.23%	90.13 ± 10.25%	78.45 ± 10.64%

The results are expressed as mean ± standard deviation from at least three independent experiments. TFC (total flavonoid content) is expressed as quercetin equivalents (µg/mg extract). TPC (total polyphenol content) is expressed as gallic acid equivalents (µg/mg extract). TAC (total antioxidant capacity) and FRPA (iron-reducing power) are relative to ascorbic acid. DPPH activity is reported as inhibition percentage at 1.25 mg/mL.

## Data Availability

The original contributions presented in the study are included in the article/Supplementary Materials; further inquiries can be directed to the corresponding authors.

## Supplementary Materials

The following supporting information can be downloaded at: https://www.mdpi.com/article/10.3390/life14121702/s1, Figure S1. FTIR spectrum from ethanolic extracts of *A. muricata* leaves from different geographic zones. Figure S2. Thin-layer chromatography (TLC) analysis of the ethanol extract and infusion of *Annona muricata* and reference compounds. Table S1. ∆E values for the overall color difference (∆E^2^) and post hoc test results for the anti-browning assay of apple slices treated with *Annona muricata* ethanolic extract and infusions.
