# Supplementary material for: Comparative Analysis of Infusions and Ethanolic Extracts of Annona muricata Leaves from Colima, Mexico: Phytochemical Profile and Antioxidant Activity
Source: Life (Basel). 2024 Dec 23;14(12):1702. doi: 10.3390/life14121702 (PMC11677062; doi:10.3390/life14121702)
Supplement: Supplementary file 1 [file life-14-01702-s001.zip › life-3360436-supplementary.pdf]

Suplementary files

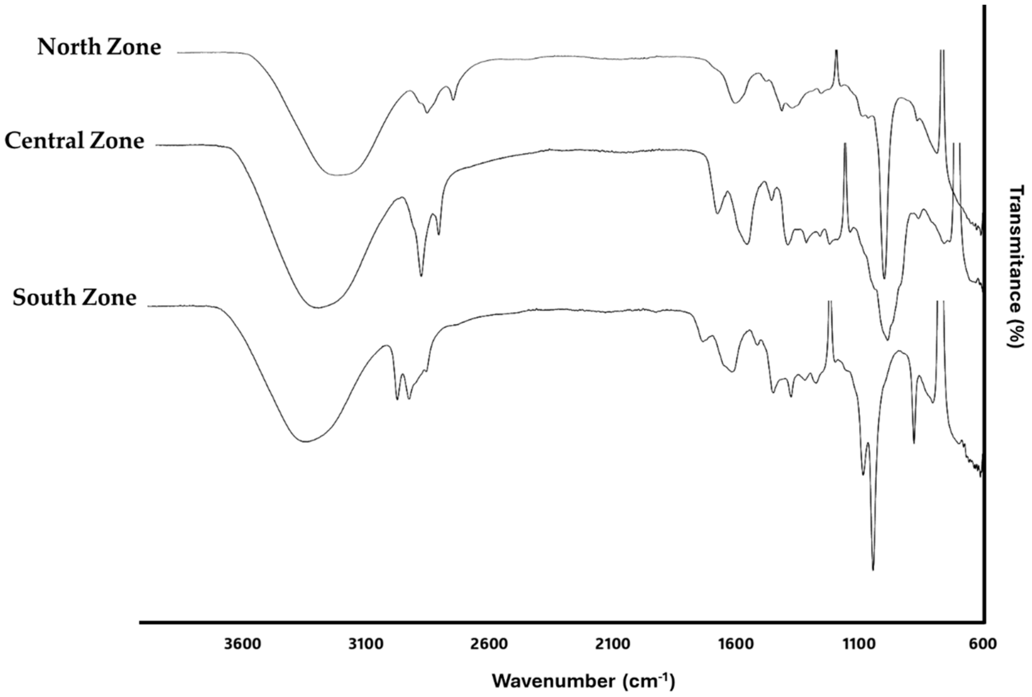

Figure S1. FTIR spectrum from ethanolic extracts of *A. muricata* leaves from different geographic zones

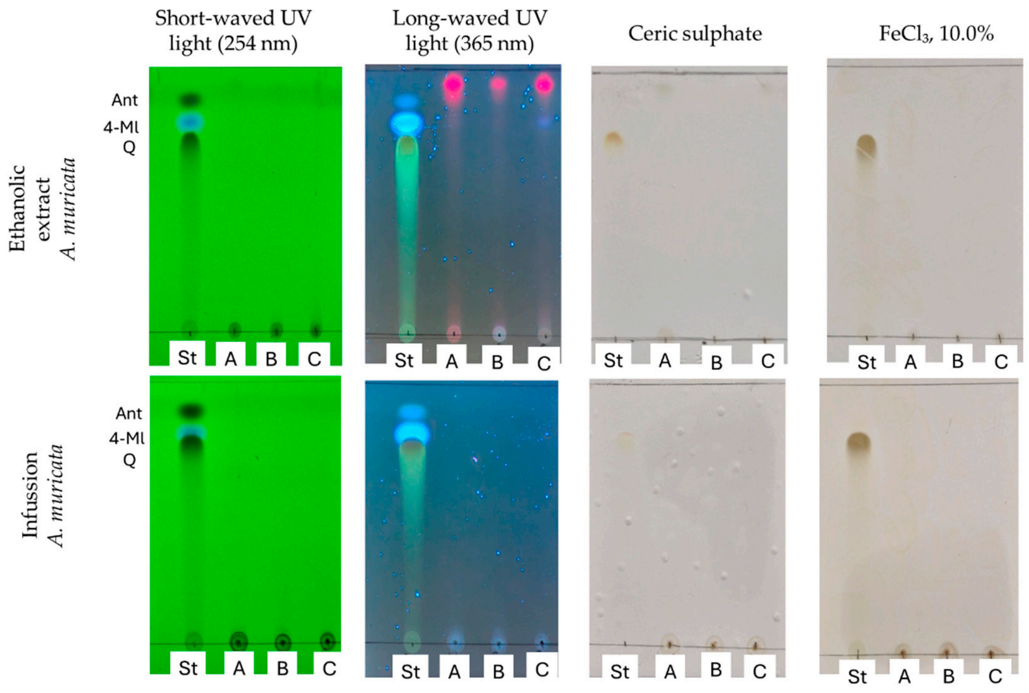

Figure S2. Thin-layer chromatography (TLC) analysis of the ethanol extract and infusion of *Annona muricata* and reference compounds. The figure presents four TLC plates based on visualization methods: chromatography under short-waved UV light (254 nm), chromatography under long-waved UV light (365 nm), visualization with ceric sulfate, and visualization with 10% ferric chloride ($FeCl_3$, 10.0%). The samples analyzed include A (sample from the north zone, 5 mg/mL), B (sample from the central zone, 5 mg/mL), C (sample from the south zone, 5 mg/mL), St (Standard: quercetin, 1 mg/mL, Rf: 0.80), Ant (anthrone, 1 mg/mL, Rf: 0.95), and 4-ML (4-methylumbelliferone, 1 mg/mL, Rf: 0.85). The solvent system used for chromatography was 99% AcOET:1%MeOH.

Table S1. ΔE values for the overall color difference (ΔE^2) and post hoc test in the antibrowning assay of apple slices treated with *Annona muricata* ethanolic extract and infusions.

Groups			Control	Ascorbic acid	Infusion			Ethanolic extract		
					North Zone	Central Zone	South Zone	North Zone	Centra l Zone	South Zone
	**Hour 24**	**Media±SEM**	**post Hoc P-values on hour 12**							
	Control	16.25±3.08		0.0150	0.0231	0.0228	0.0953	0.0948	0.2574	0.0869
	Ascorbic acid	5.98± 0.35	0.0150		0.8999	0.8999	0.8999	0.8999	0.7337	0.8999
Infusion	North Zone	6.54±1.87	0.0231	0.8999		0.8999	0.8999	0.8999	0.8509	0.8999
	Central Zone	6.52±2.06	0.0228	0.8999	0.8999		0.8999	0.8999	0.8477	0.8999
	South Zone	8.66±1.59	0.0953	0.8999	0.8999	0.8999		0.8999	0.8999	0.8999
Ethanolic extract	North Zone	8.43±0.36	0.0948	0.8999	0.8999	0.8999	0.8999		0.8999	0.8999
	Central Zone	9.94±1.61	0.2574	0.7337	0.8509	0.8477	0.8999	0.8999		0.8999
	South Zone	8.31±1.82	0.0869	0.8999	0.8999	0.8999	0.8999	0.8999	0.8999	
	P (ANOVA)	<0.001								
	**Hour 48**	**Media±SEM**	**post Hoc P-values on hour 24**							
	Control	21.73±1.91		0.0010	0.0126	0.0010	0.0010	0.0147	0.8999	0.4469
	Ascorbic acid	8.61±1.94	0.0010		0.7969	0.8999	0.8999	0.7543	0.0016	0.0409
Infusion	North Zone	12.00±1.93	0.0126	0.7969		0.4432	0.4432	0.8999	0.0272	0.4764
	Central Zone	7.06±1.03	0.0010	0.8999	0.4432		0.8999	0.3999	0.0010	0.0124
	South Zone	7.50±2.25	0.0010	0.8999	0.4432	0.8999		0.5000	0.0010	0.0178
Ethanolic extract	North Zone	12.19±1.00	0.0147	0.7543	0.8999	0.3999	0.5000		0.0318	0.5191
	Central Zone	20.81±1.31	0.8999	0.0016	0.0272	0.0010	0.0010	0.0318		0.6581
	South Zone	16.80±1.28	0.4469	0.0409	0.4764	0.0124	0.0178	0.5191	0.6581	
	P (ANOVA)	<0.001								
	**Hour 72**	**Media±SEM**	**post Hoc P-values on hour 36**							
	Control	21.15±2.79		0.0010	0.1787	0.0146	0.2304	0.1473	0.0054	0.0010
	Ascorbic acid	7.55±1.06	0.0010		0.1334	0.7434	0.1015	0.0010	0.0010	0.0010
Infusion	North Zone	14.56±2.49	0.1787	0.1334		0.8491	0.8999	0.0010	0.0010	0.0010
	Central Zone	11.29±1.19	0.0146	0.7434	0.8491		0.7654	0.0010	0.0010	0.0010
	South Zone	14.94±1.05	0.2304	0.1015	0.8999	0.7654		0.0010	0.0010	0.0010
Ethanolic extract	North Zone	28.03±0.79	0.1473	0.0010	0.0010	0.0010	0.0010		0.6432	0.1525
	Central Zone	32.23±1.99	0.0054	0.0010	0.0010	0.0010	0.0010	0.6432		0.8999
	South Zone	34.85±0.95	0.0010	0.0010	0.0010	0.0010	0.0010	0.1525	0.8999	
	P (ANOVA)	<0.001								

Media ± SEM of ΔE values for the overall color difference (ΔE^2) in the antibrowning assay of apple slices treated with *Annona muricata* ethanolic extracts and infusions. All treatments were tested at a concentration of 0.5 mg/mL, with distilled water serving as the control and ascorbic acid (0.5 mg/mL) as a reference. The browning of freshly cut apple slices was evaluated over 72 hours at 20°C. Statistical significance was determined using Tukey's post hoc test at a significance level of 0.05. Each storage time point includes photographs of apple slices treated with *Annona muricata* ethanolic extract at 0.5 mg/mL as well as infusions from different geographic zones (north, central, and south). Ascorbic acid (0.5 mg/mL) was used as the positive control for antioxidant activity. All samples were prepared in aqueous solutions.
